# Experimental Barley Flour Production in 12,500-Year-Old Rock-Cut Mortars in Southwestern Asia

**DOI:** 10.1371/journal.pone.0133306

**Published:** 2015-07-31

**Authors:** David Eitam, Mordechai Kislev, Adiel Karty, Ofer Bar-Yosef

**Affiliations:** 1 Independent researcher, Hararit, Israel; 2 Faculty of Life Sciences, Bar-Ilan University, Ramat-Gan, Israel; 3 Independent researcher, Zikhron Ya'akov, Israel; 4 Department of Anthropology, Harvard University, Cambridge, Massachusetts, United States of America; Murdoch University, AUSTRALIA

## Abstract

Experimental archaeology at a Natufian site in the Southern Levant documents for the first time the use of 12,500-year-old rock-cut mortars for producing wild barley flour, some 2,000 to 3,000 years before cereal cultivation. Our reconstruction involved processing wild barley on the prehistoric threshing floor, followed by use of the conical mortars (a common feature in Natufian sites), thereby demonstrating the efficient peeling and milling of hulled grains. This discovery complements nearly 80 years of investigations suggesting that the Natufians regularly harvested almost-ripe wild cereals using sickles hafted with flint blades. Sickles had been replicated in the past and tested in the field for harvesting cereals, thusly obtaining the characteristic sheen along the edge of the hafted flint blades as found in Natufian remnants. Here we report that Natufian wide and narrow conical mortars enabled the processing of wild barley for making the groats and fine flour that provided considerable quantities of nourishment. Dishes in the Early Natufian (15,000–13,500 CalBP) were groat meals and porridge and subsequently, in the Late Natufian (13,500–11,700 CalBP), we suggest that unleavened bread made from fine flour was added. These food preparing techniques widened the dietary breadth of the sedentary Natufian hunter-gatherers, paving the way to the emergence of farming communities, the hallmark of the Neolithic Revolution.

## Introduction

The Neolithic Revolution in southwestern Asia is considered to have taken place some 11,700–10,500 years ago when sedentary hunter-gatherers adopted the cultivation of cereals and legumes [1]. Although hunting and gathering continued, significant efforts were also devoted to plant food production [2]. Most, but not all, investigators accept the observation that the “Domestication Syndrome” was fully achieved about 10,500 years ago, following a thousand years of systematic cultivation along with the adoption of goat, sheep, pig and cattle husbandry [3]. Here we describe experiments that demonstrate how the production of cereal groats and fine flour from collected wild barley (*Hordeum spontaneum*) already took place during the Late Natufian culture, some 12,500 years ago, roughly 2000 to 3000 years before the onset of the Neolithic Revolution. Apparently, this technical development enabled the enhanced consumption of unleavened barley bread, a possible major product that could be made at that time from flour; other flour-based meals would have required portable vessels or ovens, not found in Natufian sites. However, one cannot firmly rule out the possibility that flour was consumed per se (but see S1 Text).

**Fig 1:**
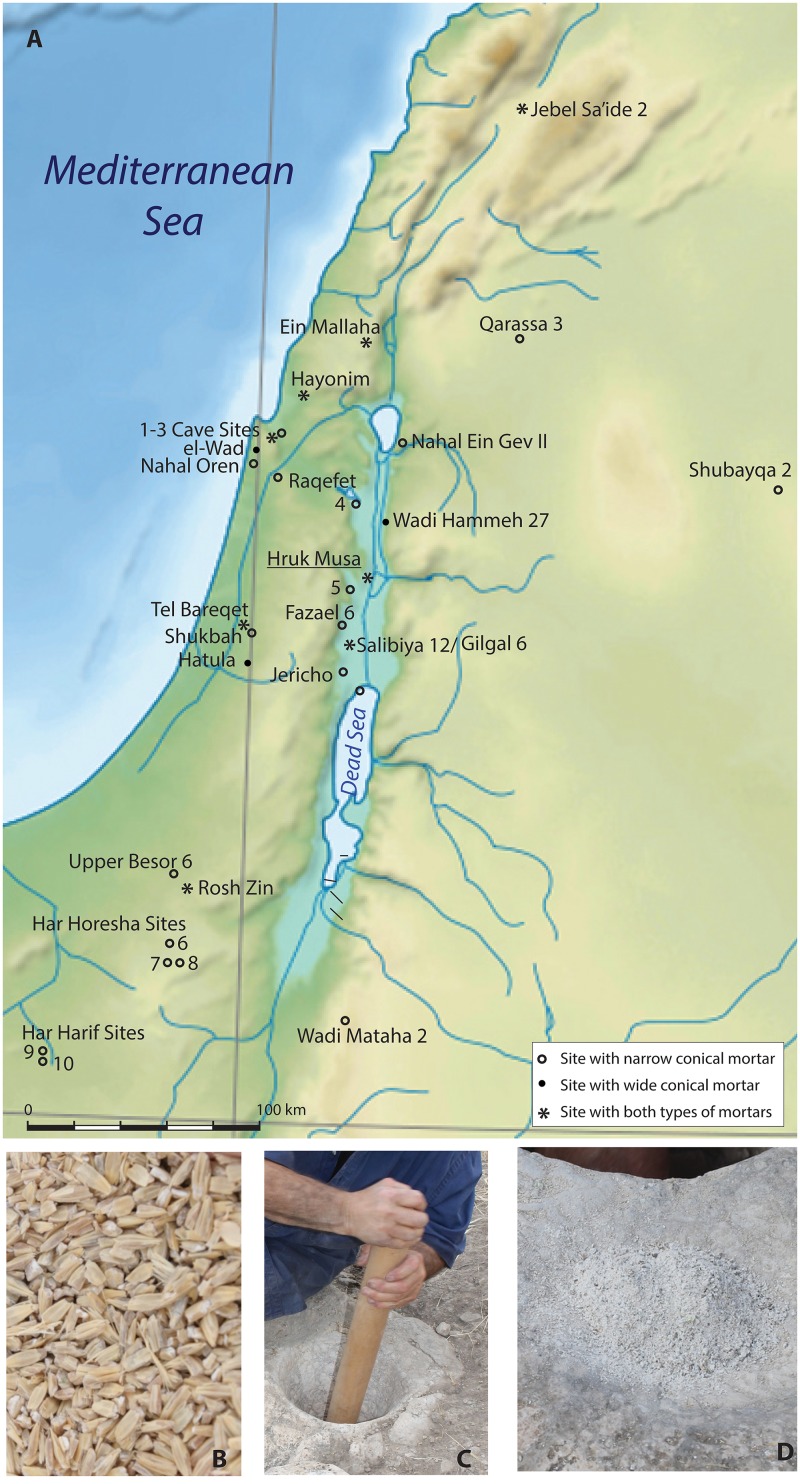
A. Map of Natufian sites with narrow and wide conical mortars in the Southern Levant: (1) Small cave sites 11, 17, 22; (2) Ornit Cave; (3) Usba Cave; (4) Wadi Malich Terrace; (5) Umm Sawaneh 5; (6) Rosh Horesha; (7) Saflulim; (8) Romam; (9) Abu Salem; (10) Ramat Harif; background image © Sémhur / Wikimedia Commons / CC-BY-SA-3.0. B. Almost peeled barley grain subsequent to the third shift of dehusking in the narrow conical mortar. C. Grinding the dehusked grains to flour by intensive radial motion in a narrow conical mortar with a long wooden pestle. D. Fine flour and some groats deposited in the adjacent cuphole, subsequent to the first shift of milling.

Thousands of hand-made hollows cut into bedrock or stone blocks were discovered in numerous scattered sites throughout the Southern Levant (Fig 1A, [4, 5]).

These hollows were assumed to have been generally used for processing plant food, but their specific uses and products were never demonstrated. In order to examine the possible use of the various conical mortars, of which 269 were found in Natufian sites, we first documented a total of nearly a thousand rock-cut utensils from 30 such sites in the region (S1 Table and S1 Fig). Their potential uses were examined through a survey of historical and ethnographic parallels. A comprehensive classification system of stone utensils, including rock-cut installations and ground stones, was created and detailed descriptive studies were carried out [6] (compare this with a study based on 111 "bedrock features" in two sites) [7]. Subsequently we selected a few of these rock-cut utensils for systematic experiments to examine their possible use in grain processing.

For this purpose, we harvested large amounts of fully ripe wild barley, the only large-grain wild cereal that grew extensively in the Southern Levant during the Terminal Pleistocene and Early Neolithic periods. Barley seeds rarely occur in Late Pleistocene sites, such as the water-logged site of Ohalo II [8], but they are abundant in early Holocene sites in the lower Jordan Valley [9–13]. However, barley grains are not edible unless their sharp awn bases are removed. Without awns, the grains—still coated by hulls—can be eaten plain as coarse, fine groats or coarse flour, as well as porridge. But for making flour for bread, the husks must also be removed, as clearly evident by the standard threshing of cereal grains prior to milling. This step is essential because the chaff in milled hulled grain prevents dough preparation. Dehusking barley spikelets requires fully ripe, hard grains for peeling off the adhering hulls. Wild barley grains are angular as compared to the rounded cultivated ones and are consequently less easily peeled (S2 Text).

The Natufian rock-cut utensils are devices, similar to mobile ground-stone implements such as mortars and carved stone goblets (as well as milling utensils, bowls and basins, S1 Table and S1A Fig). Here we describe two types of conical mortars of different shapes recorded in Natufian sites (S1 Table, S1C, S1D, S1F and S1G Fig): (a) a wide conical mortar with 60° sloping sides (n = 53) and (b) a deep and narrow conical mortar with a diameter of 20 cm and sloping sides of 20° (n = 216). The first type was recovered in both Early and Late Natufian sites and is directly associated with threshing floors. The narrow conical mortars were recovered exclusively in Late Natufian sites [6, 14].

Narrow conical mortars have pointed inner bases. They were, therefore, not designed for the crushing and pounding of nuts, such as acorns [15]: the mash would have been difficult to extract from the narrow mortar bottom. Nor are they suitable for brewing or for holding or storing liquids because of their small volume (4 liters) and the difficulty of extracting even half of the mortar volume [16]. About 95% of the narrow conical mortars were uncovered in domestic contexts without demonstrable cultural values apart from food preparation. Suggestions that the mortars represent hierarchic masonry competitions or that they symbolize sexual organs or intercourse are not supported by additional evidence and therefore cannot be verified [17]. A few bottom-pierced mortars (some 9%) seem to have been caused by intensive use (S1C Fig; as seen also in querns [18]) or through water dissolution. Some of these holes were repaired by a fitted pebble. In certain cases, they served as physical markers for burials such as in Nahal Oren Terrace [14, 19, 20].

Historically, the same kind of mortars with a conical or strongly curved narrow parabolic inside-surface were used until recently in Turkey and other countries for dehusking wheat, dehulling barley and peeling off rice bran, while preserving the whole grains [21, 22]. In addition, the inner walls of the Natufian conical mortars show clear marks of intensive vertical (S1C Fig) and radial motions of a long pestle, probably made of hard wood.

We undertook an experimental investigation at the Late Natufian site of Huzuq Musa (also named Hruk Musa, S1 Table) in the southern Jordan valley (S3A Fig), where rock-cut utensils were readily available [14, 23]. Three utensils were used in this experiment: a threshing floor (namely a flat rock surface), an accompanying wide conical mortar and a narrow conical mortar (S1B, S1B:2 and S1D Fig).

## Materials and Methods

The archaeological survey of Huzuq Musa by David Eitam (2003–2006, 2013) and of the other Natufian sites were permitted by the archaeological officer of the Civil Administration, licenses 1164, 1115 and the Israel Antiquities Authority, licenses G-16/2007, S-174/2010. The experimental operations were permitted by the archaeological officer of the Civil Administration, license 1166. All precautions were taken during the experimental study to avoid any damage to the three (out of 61) prehistoric utensils. No specific permissions were required for these locations and activities as it was part of the survey licenses. The field studies did not involve endangered or protected species.

Four guidelines were drawn up for accomplishing a successful experiment:

1. The experiment is to be conducted at the site using the actual Natufian utensils. This approach enabled us to reconstruct the utilization of the implements by their ancient manufacturers in the order dictated by their respective locations at the site, a goal inconsistent with the use of replicas of the different utensils (see Guideline 4 below). Use of the prehistoric utensils ensured that the findings were not distorted by unknown variables that could be introduced by the construction and use of replicas.
2. The work is to be designed to follow the steps of processing as suggested by the archaeo-industrial complex. Thus the different utensils at a single location were systematically operated in order to reconstruct the operational sequence (*chaîne opératoire*) for obtaining the desired product.
3. Because the multiple variables involved in the operation could not be replicated perfectly, we do not carry out a comparative operational sequence of dehusking wild barley ears in a narrow conical mortar with that in a standard concave mortar. Previous experimental and ethnoarcheological studies focused on the processing of emmer wheat and could not assist us in our study [24, 25, 26].
4. To avoid unnecessary complications, the experimental operation is to be performed using fully ripe grain and a single, simple grain-processing approach. The historic and ethnographic processing methods—namely, the rubbing and roasting of the ears or the soaking and sun-drying of the ripened or semi-ripened grains (S1 Text)–were not adopted prior to threshing, hummeling (removal of the awn base) and dehusking.

Our wooden pestle was designed according to a sample recovered from excavations at el-Amarna (Egypt), a device also recorded by a scene from a tomb of Thebes, dated to the 20th century BC [26, 27]. We made the pestle from hard wood with a short pointed end; its length and diameter were adjusted to the depth and width of the mortar to leave a narrow space between the wood and the walls (S2 Text).

### The experiment

**Fig 2:**
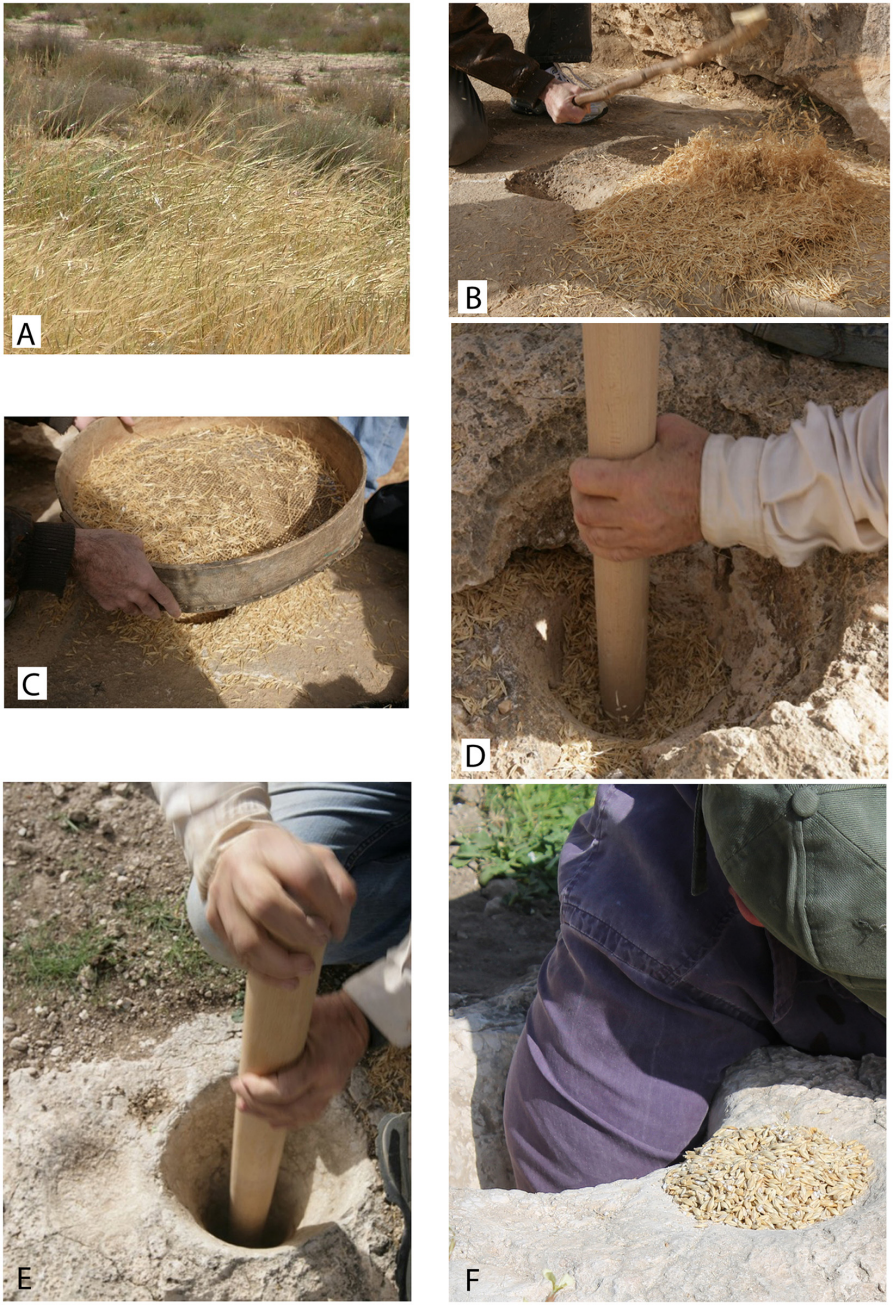
Steps of the experimental procedure using the Natufian utensils. A. A field of ripe wild barley ears with whole spikes before harvest, Negev, Israel. B. Beating spikelets on Threshing Floor II, by a heavy, bent branch with some straw, gathered in a heap. C. Sieving spikelet awns and straw using a large-hole traditional Arab sieve (sarod in Arabic). D. Hummeling spikelets with a long wooden pestle in a wide conical mortar (broken). E. Dehusking hulled spikelets with wooden pestle in a narrow conical mortar by vertical strokes (see adjacent cuphole). F. Dehusked grain and groats (after first shift of dehusking) scooped by hand from the deep mortar and deposited in the adjacent cuphole.

The following steps represent the experimental procedure (Figs 1B–1D and 2; S1B Fig):

(A) Harvesting: Cutting wild cereals with a sickle requires that the stalks are somewhat green and the ears are still unripe, thereby preventing the ear from shattering into spikelets, requiring hand gathering. Gathering ripe wild barley could be improved by laying the green stalks on the ground, close together. Subsequent trampling of the stalks allowed the grains to ripen with no scatter by the wind. The spikelets were then gathered into a basket. When ears were recumbent, instead of upright, the amount of spikelets (with some straw) gathered per hour by a single worker could be increased from 50 to 75 liters. (B) Threshing was carried out by beating them on the threshing floor with a curved stick. Spikelets were then separated with a large-hole sieve. Threshing 24 liters took 25 minutes and was achieved by three cycles of beating and sifting. This left 7.2 liters of spikelets without most of their awns (S2B Fig). (C) Hummeling (removal of the awn base) was carried out after transferring the spikelets to the adjacent wide conical mortar and pounding them with the wooden pestle for 15 minutes. At this point, the tiny sharp awn bases had been almost completely removed (about 90%) along with some of the spikelet bases (compare S2B and S2C Fig; S1 Text). Following hummeling, the spikelets were sieved with a fine sieve, which gave 5.2 liters of hulled grains still with their fused husks. (D) Dehusking took place in the narrow conical mortar, which was filled to one third of its volume (1.3 liters). The same wooden pestle was used with a routine rhythm of two gentle, controlled vertical strokes per second and the addition of some hoeing motions. In 30 minutes, the grains were almost completely dehusked (90%), with half of the grains being broken into two or three pieces (Fig 1B, S2D and S2E Fig). The chaff bits were removed by fine sieving and winnowed by gentle blowing. The processing of 1.3 liters of hulled grains produced 1,000 cc of grains and groats (S2 Text). (E) Milling was done by returning the dehusked grains and groats to the narrow conical mortar, where they were ground to flour by pushing the pointed end of the pestle to the bottom of the mortar and using intensive radial fast motions and some pounding to grind the grains against the mortar walls. It took 15 minutes to produce 230 cc of fine flour.

We found that dehusking and milling could be greatly aided by the small, round, shallow depressions frequently observed adjacent to the conical mortars (and to other utensils). These cupholes could be used appropriately to deposit material produced in the mortar by repeated hand-scooping from its bottom (Fig 2E and 2F). Small, thick pita bread could be made of unleavened dough baked on wooden coals and covered with hot embers (S1 Text). After a short baking period and turning the bread several times, the experimental loaf was ready to eat. The option of making porridge from hulled groats, as in the Early Natufian, was also available using rock-cut bowls found in Huzuq Musa and elsewhere (S1A:2 Fig).

Could the stone-utensil produced flour and the consequent baking of bread represent the provision of a staple in the diet of the hunter-gatherers of Huzuq Musa? As a hypothetical dietary requirement for a Natufian inhabitant, we assume the historic supply of four modii, about 35 liters, of grain given to a Roman worker during the winter period (about 300 cc per day) [28]. From its size (a ~0.5 hectare site with some 30 huts, S3 Fig), Huzuq Musa is estimated to have had a population of about 100, whose inhabitants would require 30 liters of grain per day. According to our experiment, the four large threshing floors discovered near the site could have supplied this quantity of processed barley. Moreover, the 31 narrow conical mortars found there have the capacity to process this amount of dehusked grain and flour in about one shift if all the mortars were working concurrently.

## Conclusions

The Late Natufian ability to prepare flour in a significant quantity and quality may well point toward the production of unleavened bread, as this was a possible major product that could have been made from flour in the Late Epipaleolithic. Other meals (such as pancakes and donuts) could not have been prepared without portable vessels or ovens.

The possibility that the Natufians consumed flour per se cannot be firmly ruled out; still we see it as being inconsistent the frequent appearance of the narrow conical mortar—which we have experimentally demonstrated to be an efficient peeling device—in Late Natufian sites, as flour can be eaten per se without de-husking the grains.

It should also be stressed that the invention of bread was a significant dietary and nutritional advance that greatly improved human food quality. Bread was and still is a major source of grain nutrition (much more than flour, groats or porridge). Bread is a good source of carbohydrates and other nutrients and is easily digested; it can also be carried as provisions. Numerous sickle blades with typical gloss resulting from harvesting cereals have been recorded [29] in more than 80 years of research of Natufian sites; they have been replicated successfully in field experiments [30]. Thus, sickles, threshing floors, conical mortars and milling utensils (S1 Fig) [31] were significant inventions of the Natufian culture, all essential for exploiting wild cereals as an important source of carbohydrates.

The technological advance from wide- to narrow conical mortars represented a major dietary change (S1A:1, S1F, S1C and S1D Fig). Meals and gruel made of husked or partly peeled ripe or unripe grains in the Early Natufian were supported in the Late Natufian by the production of flour from dehusked ripe grains and consequently the baking of unleavened bread. The dietary change from groat meals to barley bread, which probably occurred during the Late Natufian seems to have become wide spread in small villages such as Huzuq Musa and Qarassa 3 [32]. In fact, we estimated that the Huzuq Musa grain processing machinery could have produced sufficient barley bread to feed the site’s estimated inhabitants.

The development of the narrow conical mortar facilitated Late Natufian people to produce what has become the Western world's most widespread staple food. The innovation of bread-making naturally followed the long-term consumption of dishes containing mainly small grains with the addition of some hulled large-grained barley [8]. With the development of a new agro-technological system, including threshing floors, peeling utensils and milling devices, the Natufians bequeathed to their Neolithic successors a technical advancement that contributed to the establishment of agricultural societies.

## Supporting Information

S1 Fig Natufian stone utensils mentioned here. A. Small, oval-shape rock-cut threshing floor with adjacent wide conical mortar (1) and four deep bowls (2) in Early Natufian el-Wad Terrace. B. Large Threshing Floor II of Huzuk Musa with accompanying wide conical mortar (1) and milling utensils (2.) C. Narrow conical mortar cut in boulder (broken in half) from the Late Natufian Huzuk Musa (note funnel shape of the mortar's upper part and pierced bottom). D. Narrow conical mortar and adjacent cuphole cut in bedrock in the Late Natufian Huzuk Musa. Note the funnel shape of the mortar's upper part (S2 Text) and the narrow-shaft shape of the mortar's end (caused by intensive use of a long pointed pestle). E. Milling utensil in Threshing Floor II of Late Natufian Huzuk Musa (see also S1B:2 Fig); this utensil served as an additional grinding device (accounting for 2% of milling devices, the rest being the narrow conical mortars). F. Wide conical mortar cut in bedrock in Early Natufian el-Wad Terrace (see also S1A Fig). G. Wide conical mortar, goblet-vessel from Hayonim Cave, Early Natufian, phase 1, grave VIII.
(TIF)

S2 Fig Spikelets and grains of wild barley during the experimental study. A. A spikelet of wild barley after the first shift of threshing, dorsal view. The awn is cut in half. B. A spikelet of wild barley after threshing and before hummeling, dorsal view; the awn is almost removed. C. A fertile spikelet of wild barley after hummeling, dorsal view; the awn is completely removed (compare B to C). D. A wild barley grain during the dehusking phase, ventral view; the lemma (outer, dorsal husk) and parts of the palea (inner, ventral husk) are removed. E. A piece of wild barley grain after dehusking, ventral view; the husks are completely removed. Photo by M. David.
(TIF)

S3 Fig Map of Late Natufian Huzuq Musa with well-preserved surface architectural remains and rock-cut utensils. Huzuk Musa is a large single-period, Late Natufian (0.5 hectare) site in the southern Jordan Valley. About 30 round huts were located above surface: 18 stone-wall huts are situated in the north and center of the site (III A-J, N-T, respectively), while three huts are located outside the terraced wall adjoining large boulders (V; not drawn on map). Other similar-sized structures (marked by different recent flora) are placed in between the stone-wall huts (IV), and a central large structure (VII, divided into two spaces, K and L), adjoins a law cliff. The site is surrounded by a long terrace wall in the east (double line X1-X, Y, and a second line combined with large boulders Z, W, V, built of large square stones). One line of large boulders stands perpendicular to the terrace wall in the north, cliffs are in the west, and a cluster of large boulders and cliffs with caves are in the southwest. Two diverse zones are visible at the site: the large dwelling zone in the north, bounded by a line of boulders in the north, and additional dwelling in the center, and a 1300 square meter open space zone, lined by the terrace wall in the south. Two large flat bedrock exposures above the northern cliffs (I, II) also appear to have been used by the inhabitants, as well as the far southern threshing area (VI), evidenced by exclusive Late Natufian utensils cut in the bedrock. Most of the 61 rock-cut utensils found (some are composite utensils with several rock-cut items, see S1 Table; marked by red dots) are located near or in the dwelling structures. Four threshing floors (two of which are noticeable, VI) and some dozen accompanying utensils are located 200 m to the south of the site. Experiments were conducted in the lower Threshing Floor II, in the adjacent wide conical mortar (VI), and in the narrow conical mortar located in hut B (Surveyor A. Yamim).
(TIF)

S1 Table Catalog of four Natufian stone utensils in the southern Levant. The data presented includes the four types of utensils discussed here; it is part of a larger catalog of stone tools for processing and preparing foodstuff, as well as for storage, ritual and mortuary use. Utensils are listed within sites, from north to south and divided by a solid line. Type, typological date and location are listed in columns 3 to 5, followed by detailed physical measurements, wear of the inner surface due to use and fabrication, rock formation, and additional features (note). Most of the utensils were recorded first-hand during the study, while others, mentioned specifically, were reported by others in previous and recent studies (such as, Ozba Cave, Small Cave 17, Small Cave 11, Small Cave 22, Subayqa 2 and Qarassa 3 (see references in manuscript).
**Abbreviations**
Exokarstkarstic erosion on rock surface; all measurements are in centimeters (except for features followed by m. (meter)(#)restored/estimated measurement#/1Number of item as part of composite utensilGSground stoneRCUrock cut utensilCBcut in boulder or stone blockNCMnarrow conical mortarWCMwide conical mortarMUmilling utensils (include grinding installations and querns)TFthreshing floorCUPadjacent cuphole*typological dateNNatufianENEarly NatufianLNLate NatufianNnorthSsouthWwestEeast&andCom.complexHhumanPhs.PhaseStr.structure
(PDF)

S1 Text Traditional unleavened bread and ancient barley groat meals (DOCX)

S2 Text Technical analysis. (DOCX)
