# Supplementary figures and images for: Experimental Barley Flour Production in 12,500-Year-Old Rock-Cut Mortars in Southwestern Asia
Source: PLoS One. 2015 Jul 31;10(7):e0133306. doi: 10.1371/journal.pone.0133306 (PMC4521830; doi:10.1371/journal.pone.0133306)

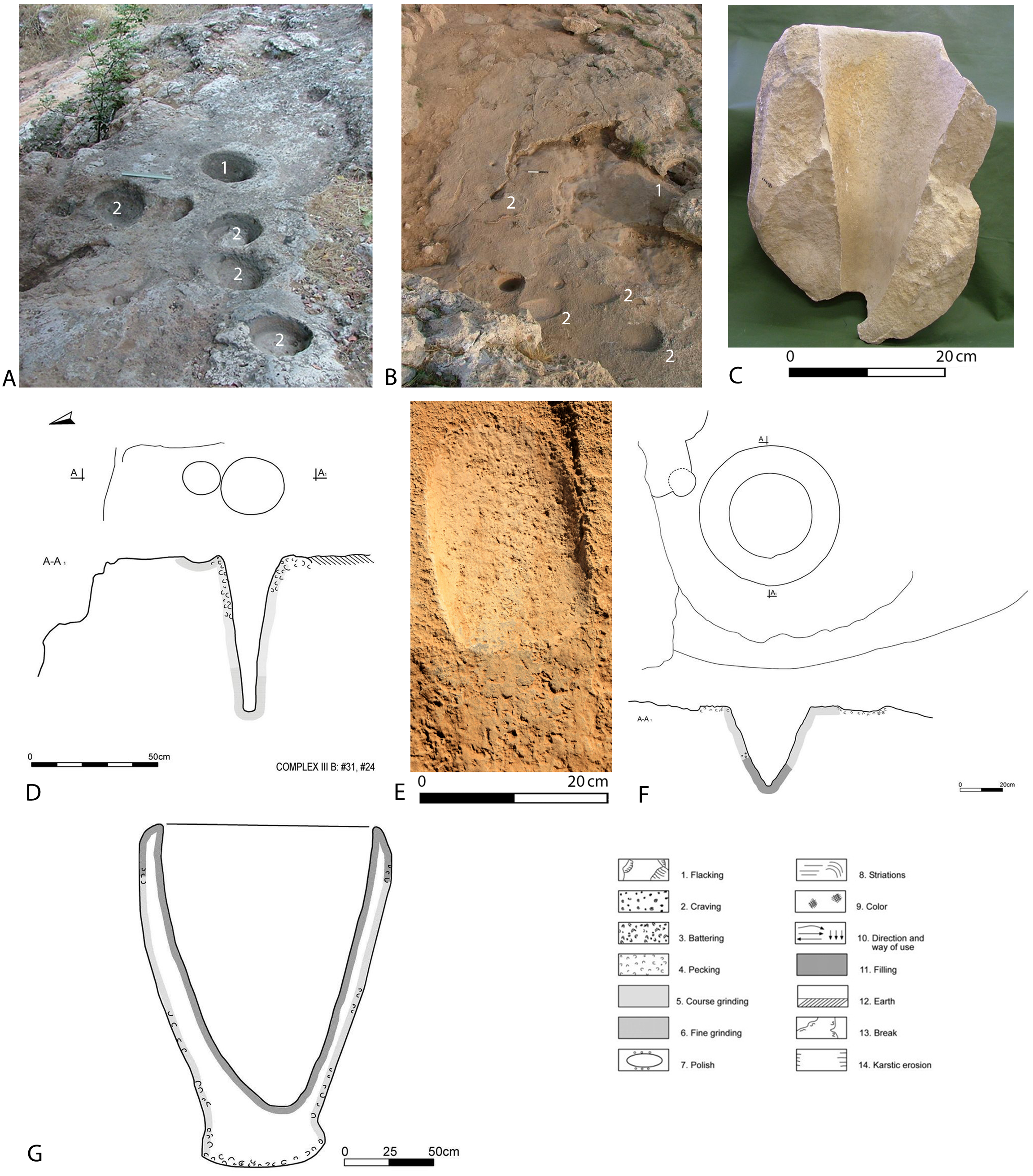

Supplement: S1 Fig — A. Small, oval-shape rock-cut threshing floor with adjacent wide conical mortar (1) and four deep bowls (2) in Early Natufian el-Wad Terrace. B. Large Threshing Floor II of Huzuk Musa with accompanying wide conical mortar (1) and milling utensils (2.) C. Narrow conical mortar cut in boulder (broken in half) from the Late Natufian Huzuk Musa (note funnel shape of the mortar's upper part and pierced bottom). D. Narrow conical mortar and adjacent cuphole cut in bedrock in the Late Natufian Huzuk Musa. Note the funnel shape of the mortar's upper part (S2 Text) and the narrow-shaft shape of the mortar's end (caused by intensive use of a long pointed pestle). E. Milling utensil in Threshing Floor II of Late Natufian Huzuk Musa (see also S1B:2 Fig); this utensil served as an additional grinding device (accounting for 2% of milling devices, the rest being the narrow conical mortars). F. Wide conical mortar cut in bedrock in Early Natufian el-Wad Terrace (see also S1A Fig). G. Wide conical mortar, goblet-vessel from Hayonim Cave, Early Natufian, phase 1, grave VIII. (TIF) [file pone.0133306.s001.tif]

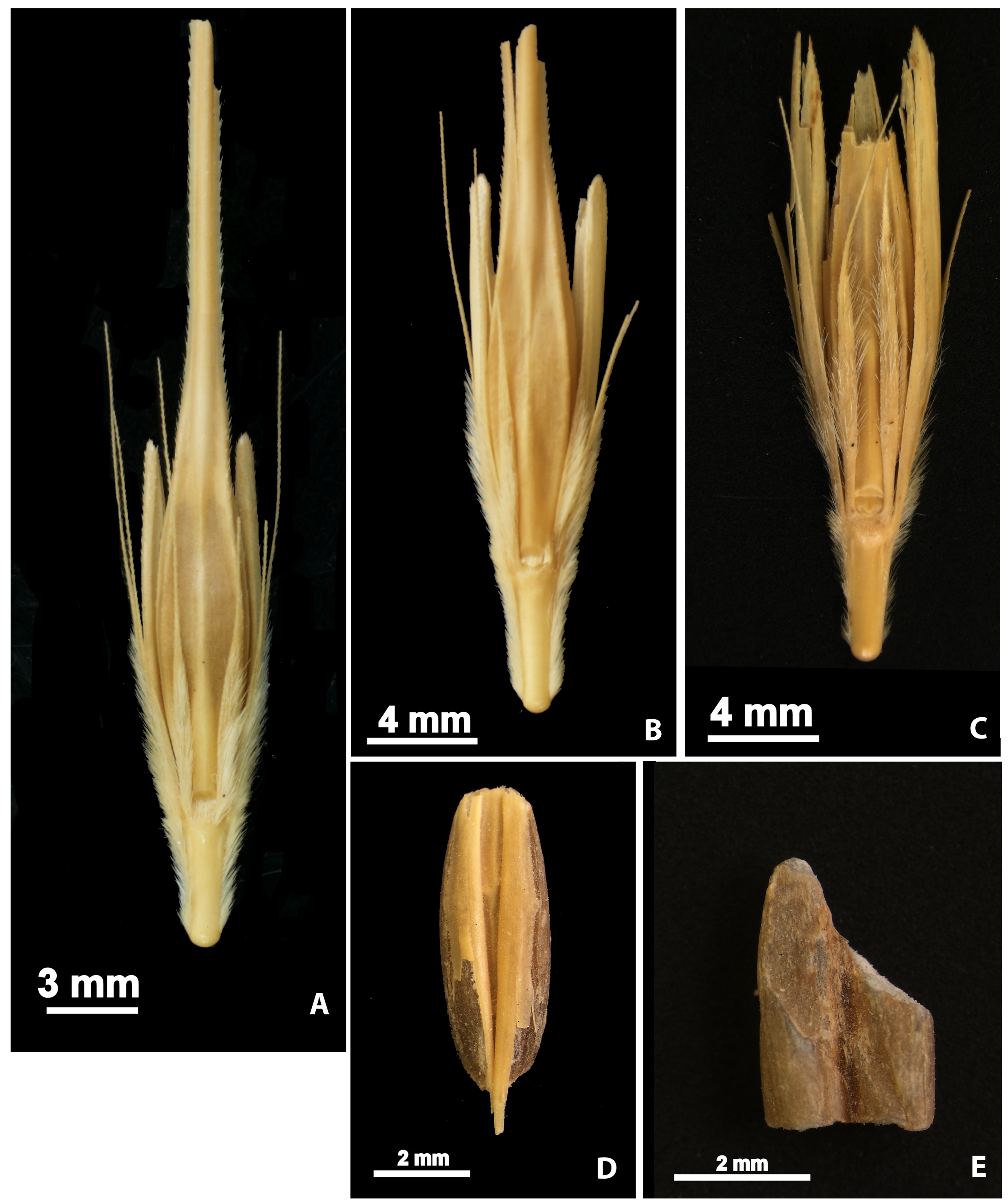

Supplement: S2 Fig — A. A spikelet of wild barley after the first shift of threshing, dorsal view. The awn is cut in half. B. A spikelet of wild barley after threshing and before hummeling, dorsal view; the awn is almost removed. C. A fertile spikelet of wild barley after hummeling, dorsal view; the awn is completely removed (compare B to C). D. A wild barley grain during the dehusking phase, ventral view; the lemma (outer, dorsal husk) and parts of the palea (inner, ventral husk) are removed. E. A piece of wild barley grain after dehusking, ventral view; the husks are completely removed. Photo by M. David. (TIF) [file pone.0133306.s002.tif]

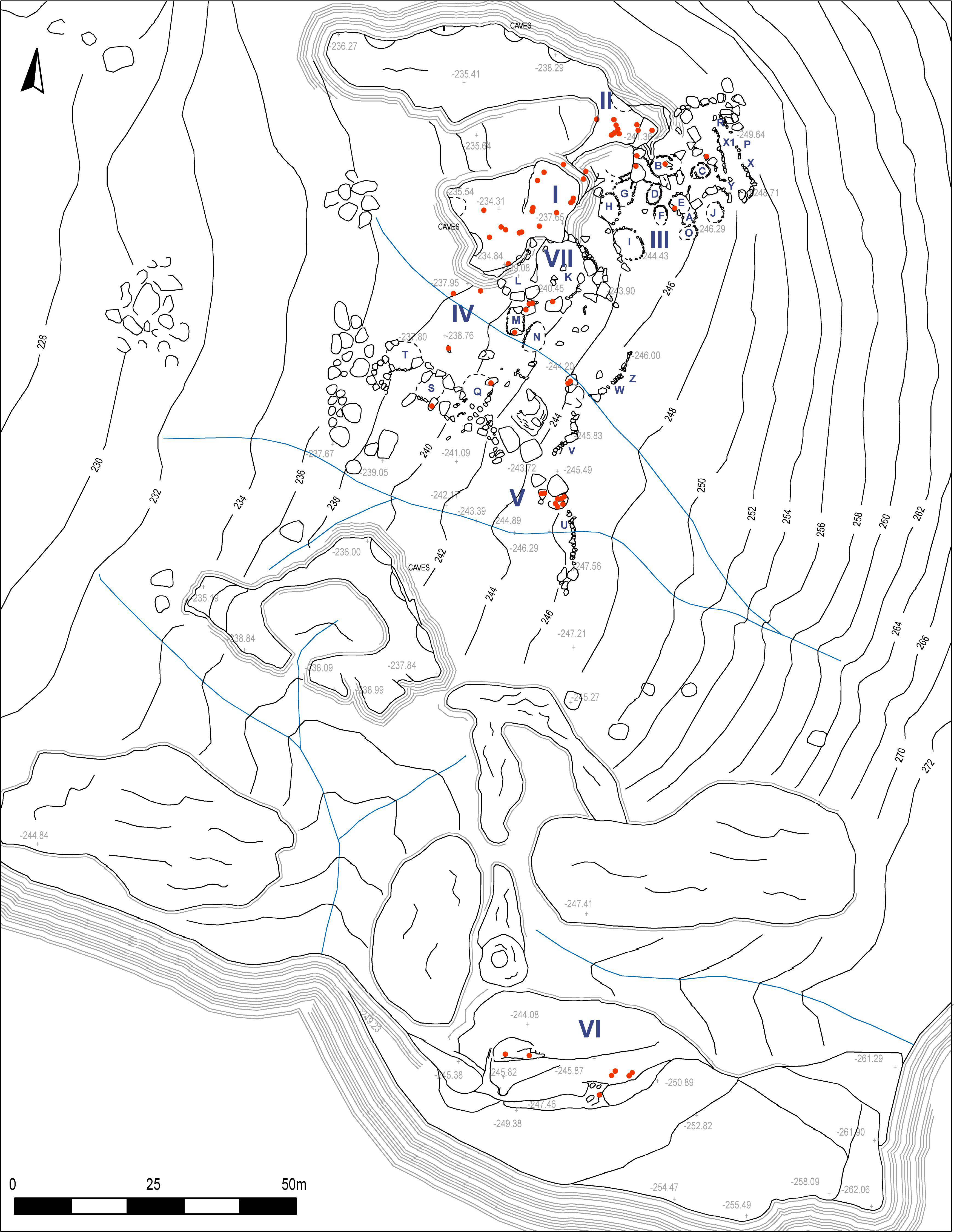

Supplement: S3 Fig — Huzuk Musa is a large single-period, Late Natufian (0.5 hectare) site in the southern Jordan Valley. About 30 round huts were located above surface: 18 stone-wall huts are situated in the north and center of the site (III A-J, N-T, respectively), while three huts are located outside the terraced wall adjoining large boulders (V; not drawn on map). Other similar-sized structures (marked by different recent flora) are placed in between the stone-wall huts (IV), and a central large structure (VII, divided into two spaces, K and L), adjoins a law cliff. The site is surrounded by a long terrace wall in the east (double line X1-X, Y, and a second line combined with large boulders Z, W, V, built of large square stones). One line of large boulders stands perpendicular to the terrace wall in the north, cliffs are in the west, and a cluster of large boulders and cliffs with caves are in the southwest. Two diverse zones are visible at the site: the large dwelling zone in the north, bounded by a line of boulders in the north, and additional dwelling in the center, and a 1300 square meter open space zone, lined by the terrace wall in the south. Two large flat bedrock exposures above the northern cliffs (I, II) also appear to have been used by the inhabitants, as well as the far southern threshing area (VI), evidenced by exclusive Late Natufian utensils cut in the bedrock. Most of the 61 rock-cut utensils found (some are composite utensils with several rock-cut items, see S1 Table; marked by red dots) are located near or in the dwelling structures. Four threshing floors (two of which are noticeable, VI) and some dozen accompanying utensils are located 200 m to the south of the site. Experiments were conducted in the lower Threshing Floor II, in the adjacent wide conical mortar (VI), and in the narrow conical mortar located in hut B (Surveyor A. Yamim). (TIF) [file pone.0133306.s003.tif]
