# Supplementary material for: Experimental Barley Flour Production in 12,500-Year-Old Rock-Cut Mortars in Southwestern Asia
Source: PLoS One. 2015 Jul 31;10(7):e0133306. doi: 10.1371/journal.pone.0133306 (PMC4521830; doi:10.1371/journal.pone.0133306)
Supplement: S1 Table — The data presented includes the four types of utensils discussed here; it is part of a larger catalog of stone tools for processing and preparing foodstuff, as well as for storage, ritual and mortuary use. Utensils are listed within sites, from north to south and divided by a solid line. Type, typological date and location are listed in columns 3 to 5, followed by detailed physical measurements, wear of the inner surface due to use and fabrication, rock formation, and additional features (note). Most of the utensils were recorded first-hand during the study, while others, mentioned specifically, were reported by others in previous and recent studies (such as, Ozba Cave, Small Cave 17, Small Cave 11, Small Cave 22, Subayqa 2 and Qarassa 3 (see references in manuscript). Abbreviations Exokarstkarstic erosion on rock surface; all measurements are in centimeters (except for features followed by m. (meter)(#)restored/estimated measurement#/1Number of item as part of composite utensilGSground stoneRCUrock cut utensilCBcut in boulder or stone blockNCMnarrow conical mortarWCMwide conical mortarMUmilling utensils (include grinding installations and querns)TFthreshing floorCUPadjacent cuphole*typological dateNNatufianENEarly NatufianLNLate NatufianNnorthSsouthWwestEeast&andCom.complexHhumanPhs.PhaseStr.structure (PDF) [file pone.0133306.s004.pdf]

Site and utensil No.	Form of utensil	Type of utensil	Date/ Typological date *	Location	Interior Measures (cm)					Interior Wear			Exterior Measures (cm)			Rock formation	Notes
					Upper axis 1	Upper axis 2	Lower axis 1	Lower axis 2	Length	Rim wear	Side wear	Lower wear	Axis 1	Axis 2	Length		
Jebel Sa'ide II	GS	**NCM**	LN	on surface	25		5		42	fine ground			39		60	limestone	Cylindrical base; measures & utensil shapes (1-7) are reconstructed.
2	vessel	**NCM**	LN	on surface	23		5		50	fine ground	fine ground	fine ground	28		59	volcanic rock	Shape: round cone, base; ext. wear: fine ground.
3	GS	**NCM**	LN	on surface	26		7		42	fine ground			39		49	limestone	Barrel shaped, round base, ext. wear: pecked ground.
4	GS	**NCM**	LN	on surface	16		4		28	fine ground			39		49	limestone	Shape: thick cylinder, round base, wear: pecked, ground.
5	GS	**NCM**	LN	on surface	18		3		40	fine ground			25		22	limestone	Barrel shape, missing base, wear: pecked, ground.
7	GS	**WCM**	LN	placed on H. burial	18		3		124				25		43	limestone	Thick cylinder, round base; wear: pecked, ground. Half item.
Ein Mallaha (Eynan) 1	vessel	**WCM**	N	Perrot Exc.	38	37	3	2	53	smooth	very smooth		47.5		62	hard limestone	Thick walls & base; wear: fine pecked, ground; rim with meander relief; cone angle 45°.
2	CB	**WCM**	EN	in 26 Str.'s center	17		2		15				40	32	(40)	limestone	Cone angle 40°; probably point bottom.
3	GS	**WCM**	N	Perrot Exc.													V shaped deep bowl.
4	GS	**WCM**	N	Perrot Exc.													V shaped deep bowl.
5	GS	**NCM**	N	Perrot Exc.													V shaped deep bowl.
8	GS	**WCM**	LN	Valla Exc.	20	11							22	16	6	limestone	V shaped deep bowl.
9	GS	**MU**	LN	Valla Exc.	17				13	ground	ground	ground	24	22	27	basalt	Ext lower axis 1, - 1cm; well-designed tool; missing back side of stepped? face.
12	GS	**WCM**	N	Perrot Exc.	23.5	17			6			fine ground				basalt	V shaped deep bowl.
14	GS	**MU**	N	Perrot Exc.													Oval; flat surface in both sections.
17	GS	**small WCM**	N	Valla Exc.	9		2.5		7				13		13	basalt	Ext. lower axis 1 – 6cm; on floor of small Str. 241.
18	GS	**MU**	LN	Valla Exc.	40	23	34	14	5			fine ground				basalt	Well-designed vessel; missing back side of working face; step working face? .
19	vessel	**WCM**	N	Perrot Exc.													Two parts of thick rim of large vessel having meander decoration.
Hayonim Cave 1	vessel	**WCM**	EN	Phs. 1, grave VIII	38.5	32.5	13		48	black sheen	smooth, vertical striation	Sheen, radial striation	43.5	37	58.3	perforated basalt	Smooth wall; concave bottom; cone angle 50°; vertical striations on side.
3	GS	**WCM**	N	inner cave	18		2		18				30		22	basalt	Round side, bottom; cone angle 45°.
4	GS	**WCM**	N	entrance, Phs. I, II	30		4		42				42		50	basalt or limestone	Smooth; round sides, bottom; cone angle 57°.
5	GS	**WCM**	N	in graves fill	16		4		16				32		34	basalt or limestone	Cone angle 35°.
6	GS	**NCM**	LN*	in graves fill	12		4		30				16		38	basalt or limestone	Cone angle 19.5°.
7	GS	**NCM**	LN*	in graves fill	14		10		36				36		40	basalt or limestone	Projecting low "waist"; cone angle 25°.
8	GS	**NCM**	LN*	in graves fill	24		10		36				40		38	basalt or limestone	Projecting low "waist"; cone angle 37°.
9	vessel	**WCM**	N	in graves fill												basalt or limestone	Base of mortar, diameter & length 5; decorated with wavy engravings.
12	vessel	**WCM**	EN	inner cave, Phs. IV												basalt	Reused of high base with conical bottom having ochre stain.
13	GS	**NCM**	N	top of L. 8, Phs. IV	12		3		22							limestone	Fragment of narrow NCM with thick walls; small cuphole cut- on break.
14	vessel	**WCM**	N	L. 6, Phs .III			6.5		.							basalt	Base diameter 12.5 cm; height 4cm; cone angle 44°.
15	GS	**WCM**	EN													limestone	Five restorable fragments from 1 locus.
16	CB	**WCM**	EN	L. 6, Phs. V												limestone	

Hayonim Terrace																
1	CB	**NCM**	LN*	in dwelling Str.	24	21	13	14	47	fine pecked, ground			50	57	limestone	Barrel-shaped; Ext. lower axis 1, – 42cm; vertically broken in 2 pieces - linked by slabs wedged in sides.
2	GS	**WCM**	EN	in dwelling Str.	23		3	4	34	fine ground			30	44	limestone	Oblong WCM, bent-in walls, thin rim.
3	CB	**WCM**	N	excavated area	24		7		40						limestone	Square boulder; 3-4 utensils were briefly examined.
4	CB	**NCM**	LN*	excavated area	15				17		fine ground				limestone	Short shaft at bottom.
5	CB	**NCM?**		near 1 & 3												Fragment (Per. Inform. D. Nadel).
Qarassa III 1-46	CB	**46 NCMs**	LN*	nearby the hats												NCMs found in a large site with at least 7 small round huts.
Ornit Cave																
1	CB	**NCM**	LN*	chamber IIA	15		4	3	(20)						limestone breccia	Upper part missing due to breakage; concave bottom.
2	CB	**NCM**	LN*	chamber II B	16		4	3	.						limestone breccia	Upper part missing; concave bottom.
Nahal Ein Gev II 1	CB	**NCM**	LN	Surface	8.7		7		25.5		fine ground	fine ground	25		basalt	Ext. lower axis 2, – 28cm; Round large NCM; utensils 1-2 were briefly examined, while 3 were noticed while touring the cliff, 2012.
2	CB	**NCM**	LN	Surface	8		2.7		(25)		liner striation	smooth	16		basalt	Ext. lower axis 2, – 26.5cm; Barrel shaped boulder; Ext. fine pecked; small shaft.
3	RCU	**NCM**	LN	on cliff above site	(16.5)		4		25							Cut in wall of rock shelf in high rock step of the cliff. Questionable.
Usba Cave																Sites in Nahal Oren.
1	RCU	**NCM**	LN*	near entrance					ca.30							
2	RCU	**small WCM?**	N		ca 15		4		ca. 15							Fabrication halted by hard stones at bottom.
3	RCU	**NCM**	LN*		ca. 25				ca. 35							Round large boulder.
Small Cave 17 1	RCU	**NCM**	LN*	on floor of cave	25				40							
1/1		CUP		beside 1	8				5							
Small Cave 11 1	RCU	**WCM**	LN*	on floor in front cave	35				20							
2	RCU	**NCM**	LN*	on floor in front cave	20				10							Fabrication halted by large hard stone in rock.
3	RCU	**WCM**	N	on floor in front cave	25				20							
4	RCU	**NCM**	LN*	on floor in front cave	20				20							Fabrication halted shortly after initiation by large hard rock in the formation.
5	RCU	**WCM**	N	on floor in front cave	25				20							
Small Cave 22																
1	RCU	**NCM**	LN*	on cave floor	12				22							Fabrication halted by hard stone in the in the formation.
2	RCU	**WCM**	N	on cave floor	20				15							A conical shaft at bottom (diameter, 7cm, length, 5cm; eroded.
3	RCU	**NCM**	LN*	on cave floor	20				43							Oblong shaft at bottom (diameter, 7cm, length, 15cm) with pointed end.
4	CB	**NCM**	LN*	on floor	25				50							Large boulder lying on its side.
1 1	RCU	**NCM**	LN*	entrance	15.5				.	smooth	pecked, ground				soft limestone	Blocked by round soft limestone (5x6) at depth of 10.
5	RCU	**NCM**	LN*	in front cave, on large rock	15	12	6	4	33		smooth	fine ground			limestone breccia	Oval opening; twisted shape due to hard breccia stones at side & bottom.
Nahal Oren																
1	CB	**NCM**	LN	in graveyard,	21	20	5		55	ground, polished	fine ground		36.5	59	hard limestone	Oval opening; fine ground shaft; pierced bottom.
2	CB	**NCM**	LN	in graveyard	21.5	22.5	5		47	ground, polished	ground	fine ground	37	50	hard limestone	Pecked, fine ground upper third of one Ext. side; pierced bottom.
3	CB	**NCM**	LN	in graveyard	19	17	3.5		62	polished	fine ground	smooth	54.5	67	hard limestone	Funnel rim, 15cm, cylindrical shaft, 7cm; side ground; pierced bottom.
4	CB	**NCM**	LN	in graveyard	21	22	5		55						hard limestone	

Nahal Oren Cave 1	RCU	**NCM**	LN	cave entrance													
3	RCU	**Small NCM**	LN	cave entrance													
4	RCU	**Small WCM**	LN	cave entrance													
5	RCU	**NCM**	LN	In front of cave													
7	RCU	**NCM**		In front of cave													
8	RCU	**Small NCM**		cave entrance													
5	CB	**NCM**	LN	in graveyard		20										hard limestone	Very large boulder.
6-15	CB	**NCM**	LN*	in graveyard													Barrel shape, well ground boulders; pierced end (2 in Dagon Museum, 7 in IAA stores, Per. Inform, D. Nadel)
el-Wad Terrace																	
20	CB	**small TF**	EN	Com. III	175	135			3		ground	ground				limestone , flint breccia	Oval straightened surface surrounded in TF by ovate line, irregular groove 1.4 m long.
20/1		WCM	EN	Com. III	37		5		39	smooth	fine ground	radial striations				limestone , flint breccia	Projecting circular ring, diameter 64cm, height 1.5cm; cone angle 45◦; small cylindrical shaft at bottom.
31-32	CB	WCM?	EN	Near el-Wad 1												limestone	(Per. Inform, D. Nadel).
el-Wad Cave																	
1	CB	small WCM	EN*	in entrance	17	15	4		14.5	carved , pecked	smooth, radial striation	smooth				limestone , flint breccia	Radial striation; short cylindrical shaft at bottom, diameter 4; reused as olive oil installation.
2	CB	**small TF**	EN*	in entrance	59	64	18	21	17							limestone	Straight-angled stone tablet, well cut & finished surface, ground with oval small CUPs.
Raqefet Cave																	
1	RCU	**NCM**	LN	Com. I	20	25			45		smooth					limestone	Interior measures & shape of utensils 1-3, 6-7, 9, 13, 23, 31-32, 44 are reconstructed from published data and personal observations.
1/1		CUP		Com. I	7											limestone	
2	RCU	**NCM**	LN	Com. I	30				40	fine ground pecked						limestone	
2/1	RCU	CUP		Com. I	3		2		1.5							limestone	
3	RCU	**NCM**	LN	Com. I	30				40	pecked , ground	rough					limestone	
6	RCU	**NCM**	LN	Com. II	27	23			55	fine ground	fine ground	fine ground				limestone	Funnel rim showed: intensive wear by use; side of mortar slanted due to hard rock in breccia.
7	CB	**NCM**	LN	large rock-cut grave; Com. II	21				39	Pecked, coarse ground	fine ground	fine ground	37		42	limestone	Boulder broken in half & pierced at bottom; shaft diameter 7cm.
9	RCU	**NCM**	LN	Com. IV						ground						limestone	
13	RCU	**NCM**	LN	Com. III												limestone	
22	RCU	**NCM**	LN	Com. I	22	22			37							limestone	
23	RCU	**NCM**	LN	Com. I	19	20			22							limestone	Mortar side slanted due to rock formation.
31	RCU	**NCM**	LN	Com. IV	16	17			19							limestone	
32	RCU	**NCM**	LN	Com. IV	13	11			13					35	20	limestone	Classification questionable.
33	RCU	**NCM**	LN	Com. IV													
34	RCU	**NCM**	LN	Com. IV													
44	CB	**NCM**	LN	Com. II, baside large grave									23		47	limestone	Briefly observed in person; boulder; pierced at end.
82	RCU	**NCM**	LN*	Com. V outside cave		15			.								The utensil was filled with modern cement.

90	CB	**NCM**	LN	few scattered fragments												(Per. Inform, D. Nadel).
Subayqa II																
1-7	CB	**7 NCMs**	LN*	2 of the mortars near Str.											basalt	Some of the mortars had CUPs.
Wadi Hammeh 27																
1	vessel	**WCM**	EN	plot XX E/H	7		1.5			fine ground	fine ground	fine ground	9	11	basalt	Ext. lower axis 1, – 8.5cm; cone angle 45.
2	vessel	**WCM**	EN	plot XX E	14		2		12	fine ground	fine ground	fine ground	16.5	17	basalt	Ext. Lower axis 1, – 13cm; Cone angle possibly 50.
3	vessel	**WCM**	EN	plot XX E	21		5		23	fine ground	fine ground	fine ground	24		basalt	
5	vessel	**WCM**	EN	plot XX E	17				20		fine ground	fine ground			basalt	
6	vessel	**small WCM**	EN													Classification questionable.
7	CB	**WCM**	EN													Eroded; large; cubic boulder.
Tel Bareqet																
3041/20	CB	**NCM**	LN*	L. 3041	15	17	4		23	coarse ground	Fine& coarse ground	fine ground			nari	Eroded, pointed shaft at bottom.
3041/21	CB	**NCM**	LN*	L. 3041	23		4		70		fine ground	fine ground			nari	Short pointed shaft at bottom; reused as a vat in EB II period, 55 of the original NCM remained.
3042/1	CB/RCU	**NCM**	LN*	L. 3042	16	14	2		40			fine ground			nari	Lower part of mortar remained due to reuse as a vat in EB II period.
3048/5	RCU	**NCM**	LN*	L. 3048	21	24	3		48		fine ground	fine ground			nari	Reused in EB II period as a collecting vat; stone wedged in bottom.
3048/6	RCU	**WCM**	N	L. 3048	23	21	4		17		coarsely pecked	fine ground			nari	Small cylindrical shaft at bottom; upper part of sides eroded.
3063/1	RCU	**Small WCM**	LN*	L. 3063	17		2		30			ground & fine ground			nari	Upper part cut to serve as a collecting vat in EB II period.
Wadi Malich Terrace																
1	RCU	**NCM**	LN*	at E end of site	23		9.5		31		Pecked & ground	fine ground			limestone	Upper part of one side missing; attempt to remove hard stone by reduction & abrasion.
2	RCU	**NCM**	LN*	at E side	29	30	5	7	39		Pecked & ground	fine ground			limestone	One side missing; long funnel rim, concave end.
3	RCU	**NCM**	LN*	at E side	22	23	3		71	Pecked, ground	fine ground	fine ground			limestone	Short funnel rim; long, perfect conical mortar with narrow end.
4	RCU	**NCM**	LN*	at E side	20	21	3.5		22			fine ground			limestone	Short mortar; upper part eroded; round end.
Hruk Musa																
1	RCU	**WCM**	LN	Com. I	33	25	4.5		55	carved	pecked, ground	fine ground			limestone breccia	Wide oval funnel rim, twisted shape due to crumbled rock; shaft in WCM bottom.
1/1	RCU	CUP			8		5		1.5							Eroded.
3	RCU	**NCM**	LN	Com. I	21	22.5	4		40						limestone breccia	Eroded; shaft blocked by round pebble, repairing pierced bottom.
4	RCU	**NCM**	LN	Com. I	23	25	6		17			fine ground			limestone breccia	Eroded; smooth karst surface eroded by water.
5	RCU	**NCM**	LN	Com. I	26.5	30	3		60		carved	Smooth			limestone breccia	Shape twisted due to crumbled rock; pointed shaft, cup beside.
6	RCU	**NCM**	LN	Com. I	23		3		47		ground	fine ground			limestone breccia	Cylindrical shaft, length 15; pierced through soft layer in rock.
7	RCU	**NCM**	LN	Com. I	22	8	12		11		Carved & flaked	pecked & coarse ground			limestone breccia	Reduction of hard stones, fine ground on side around the stones.
8	RCU	**NCM**	LN	Com. I	15		12		7		carved, flaked	pecked & coarse ground			limestone breccia	Reduction of hard stones, fine ground on side around the dark stones.
9	RCU	**NCM**	LN	Com. I	19	21	3		23		pecked, ground	fine ground			limestone breccia	Smooth karst surface eroded by water.
10	RCU	**NCM**	LN	Com. IV	17		6		17		pecked, ground	fine ground			limestone breccia	Smooth karst surface eroded by water.
11	RCU	**NCM**	LN	Com. IV	23	25	6	7	54		pecked , ground	fine ground			limestone breccia	Funnel rim eroded by water; field stone wedged in lower part of mortar.

12	RCU	**NCM**	LN	Com. IV	21		10		39		pecked , ground	Pecked & ground				limestone breccia	Hollowed Shaft, bottom is missing; crumbled breccia.
13	RCU	**NCM**	LN	Com. IV	20		7		30	fine ground	pecked, ground	fine radial ground				limestone breccia	Fine radial ground on rim; hard stone, reduced by pecking & abrasion.
14	RCU	**WCM**	LN	Com. IV	24	25	7		49	Pecked & ground	fine ground	fine ground				limestone breccia	
14/1	RCU	CUP	LN	Com. IV	9	7			2							limestone breccia	
15	RCU	**NCM**	LN	Com. IV	22		5		51	coarse ground	fine ground	pecked, fine ground				limestone breccia	Upper part eroded.
16	RCU	**NCM**	LN	Com. IV	17		2		17		pecked, ground	pecked, fine ground				limestone breccia	Eroded; reused in recent times as water pit covered by a stone.
17	RCU	**NCM**	LN	Com. VII	22		5		35		fine ground	Smooth				limestone breccia	Upper part eroded.
18	RCU	**NCM**	LN	Com. VII	19	21	5		30			fine ground				limestone breccia	Most parts eroded.
19	RCU	CUP			10				2								
20	RCU	**NCM**	LN	Com. VII	17	20	5.5		29							limestone breccia	
21	RCU	**NCM**	LN	Com. III	16	17.5	3		50			pecked & ground				limestone breccia	Eroded except for the shaft.
22	RCU	**NCM**	LN	Com. III	19	20	1.5		42		fine ground	Smooth				limestone breccia	Smooth; usewear patches at bottom; flat surface (35x35x6cm) mortar.
23	RCU	**NCM**	LN	Com. III	25	28	7		35			pecked & ground				limestone breccia	Eroded.
24	RCU	**NCM**	LN	Com. III	23	25	4	46	61	radial striation	pecked, ground	fine ground					Long funnel rim .
24/1	RCU	CUP			10	8			2		pecked, ground	fine ground					Shallow concave cup
25	RCU	**NCM**	LN	Com. II	19	20	9.5		16			smooth				limestone breccia	Short eroded mortar.
26	RCU	**NCM**	LN	Com. II	17		3		31	ground	coarse ground	smooth				limestone breccia	Upper part eroded; cylindrical short shaft 3cm in diameter.
27	RCU	**NCM**	LN	Com. II	18		8		18							limestone breccia	
28	RCU	**NCM**	LN	Com. I	22		4		36							limestone breccia	
29	RCU	**NCM**	LN	Com. I	16.5		4	7	24	Pecked, ground	fine ground	fine ground				limestone breccia	Most parts eroded.
31	RCU	**NCM**	LN	Com. I	(16)				20							limestone breccia	One side missing; reconstructed from basis of bottom side.
32	RCU	**MU**	LN	Com. I	65	52			3	abraded	abraded	smooth	90	65	15	hard limestone	Large quern with edge; width 15-7cm, height 3cm; dimple in center.
33	RCU	**NCM**	LN	Com. IV	19	20	5		43	pecked & ground	fine ground	smooth				limestone breccia	Long funnel rim, shaft (-length 11cm).
34	RCU	**MU**	LN	Com. II	22	37			5							limestone breccia	Face, sides eroded by exokarst; classification questionable.
35	RCU	**MU**	LN	Com. II	40	25			7							limestone breccia	Surface eroded by exokarst.
39	CB	**NCM**	LN	Com. IV (found broken)	24.4	19	4		41.2	fine ground	coarse ground	smooth	36	34	43.3	hard limestone	Ext. lower axis 1, – 20cm; upper funnel smooth, 15cm; upper mortar smooth by use; cylindrical smooth shaft; pierced end.
72	RCU	**NCM**	LN	Com. II	20	19	5.5		47	pecked, ground	fine ground	smooth				limestone breccia	Eroded upper part of mortar.
73	RCU	**NCM**	LN	Com. I	22	21	4		32							limestone breccia	Eroded sides & bottom.
60	RCU	**TF I**	LN*	Com. VI on cliff top	7.5m	4m						natural flat surface				limestone breccia	Large oblong, semi-half circle rock surface with WCM cut in its eastern end.
69/1 (59)	RCU	**WCM**		on E end of 60	35		3	2	60	pecked, ground	fine ground	Fine ground					WCM, length -25cm, above NCM, length (with shaft) 38cm, width 20cm.
63	RCU	**TF II**	LN*	Com. VI on 2nd	5.75m	2m						straightened				limestone	Rectangular rock surface with one WCM & 14 adjacent

				rock step								surface				breccia	utensils.
63/1 (62)	RCU	**WCM**	LN*	on S edge of	33		10		41	pecked, ground	fine ground	smooth					WCM deepened & halted by hard stones; broken, cracked, eroded.
63/2 (64)		**NCM**		beside & N to 63	16		4.5		24	pecked, ground	fine ground	smooth					Twisted shape due to rock type; - Straight sides, shaft at bottom.
63/3 (65)		**NCM**		beside & N to 63	30		6		54.5	pecked, ground	fine ground	smooth					Eroded funnel end; hard stone at end; shape twisted.
63/3 (66)		**MU**		Beside & N to 65	32		21		17-5			flat , rough					Oval, step in front, open back; face eroded, 1 side broken.
63/4 (67)		**MU**		beside 64	30		18		3			flat , rough					Oblong shallow utensil, back side open; eroded; classification questionable.
63/9		**MU**		on W edge of 63	25		44		2,5			fine ground, dark patch					Oval, step in front, open back; clear striation of dark shine.
63/11		**MU**		on W edge of 63	25		48		4			rough					Oval, step in front, open back; roughened by exokarst .
63/10		**MU**		on W edge of 63	24		38		4			rough					Irregular oval, open back. Roughened by exokarst .
63/17		**MU**		mid of 63	26		39		3			smooth, shin usewear					Oval, step in front; twisted by crack.
77	RCU	**TF III**	LN	3rd rock step	(3)m		(5)m					natural rock surface					Irregular shape; cut at eastern end.
77/17		**WCM**															Cut- in rock step above, 77.
77/18		**NCM**		on floor													Halted at start fabrication by hard stone; utensil not used.
77/19		**NCM**		beside 18													
78		**TF IV**	LN	on bedrock below 77								natural surface					Irregular shape; WCM cut beside.
78/21		**WCM**			20			(25)		course ground	course ground	fine ground					Round wide cone; hard stone at end; narrow shaft around stone.
Fazael VI 5	RCU	**NCM**	LN	on cliff, 74 m W to 2	13.5		8	9	33		rough	smooth				limestone	Oval shape as result of use; small hard stones at end halted fabrication.
8	RCU	**NCM**	LN	on cliff, 50 m W to 2	21		9		40	smooth		very smooth				limestone	Slanting hard stone at bottom halted fabrication.
9	CB	**NCM**	LN	surface	17				45							limestone	
10	CB	**NCM**	LN	surface	18		5		22			Smooth	30	25	26	hard limestone	Fragment of pointed bottom of mortar; estimated measures.
Shukba Cave 1	CB	**NCM**	LN	at entrance													
Gilgal VI 1	CB	**WCM**	LN*	on upper slop			2						75	55	50	limestone	Lower part of large block with wide conical bottom of mortar.
2	CB	**NCM**	LN*	on wadi bed	21.5	20.5	4.4		39	fine pecked	ground	fine ground	74	65	40	limestone	Ext. lower axis 1, – 54cm, Ext. lower axis 2, – 50cm; Concave funnel rim; pierced at bottom, 3 cups around NCM.
2/1		CUP			14	13			2			fine ground					
2/2		CUP			13	11			1.5			fine ground					
2/3		CUP			13				2			fine ground					
3	CB	**WCM**	LN*	on wadi bed	18		8		23	ground	fine ground	smooth	60	54	25	limestone	
3/1	CB	CUP			11		6		1		smooth	smooth					
Salibiya XII 1		**WCM**	N	Com. I	23	22	4		30		ground	fine ground				limestone	Mortar narrower than average.
6	RCU	**NCM**	LN*	Com. III	16	15			25							breccia	
7	RCU	**WCM**	LN*	Com. I	18		8		14.5		ground					limestone	
8	RCU	**WCM**	LN*	NE to Com. III	20				23			ground				limestone	Half boulder, similar to the one at Gilgal VI.
Umm Sawaneh V 1	RCU	**NCM**	LN*	on medium bedrock	15		ca. 5		45							limestone	

2	RCU	**NCM**	LN*	beside 1	11				(45)							Eroded (was not dag till end).
3	RCU	**NCM**	LN*	on small bedrock	19				27							Fabrication was halted due to hard stone in the rock formation.
3/1	RCU	CUP		beside 3	9				5							
4	RCU	**MU**	N	on small bedrock	22				48							2 step utensils, one beside the other.
5	RCU	**MU**	N	on small bedrock	20				50							Installation is crack along its axis. Depth 7.
Hatula 14	RCU	**WCM**	N	Com. V	18	16			14						breccia: flint, limestone	
22	RCU	**WCM**	N	Com. XII	33	20			7						breccia: flint. limestone	
27	RCU	**WCM**	N	Com. XIII	32	18			6.5						breccia: flint. limestone	
Jericho 1	CB	**NCM**	LN*	square E1					60				40	70	limestone breccia	
2	CB	**NCM**	LN*	square E1												
Rosh Zin 1	RCU	**NCM**	LN	Com. I, N of Str.	15.5		4		33	ground , pecked	smooth	smooth			Limestone	Cracked in half after long use; sides eroded.
Rosh Zin 2	RCU	**NCM**	LN	Com. I, narrow bedrock	14	15	4	4	37			coarse ground			Limestone	
26	RCU	CUP	LN	beside 2	6	6.5			3			smooth thick patina			Limestone	Pecked occurred in modern times, as indicated by no patina.
27	RCU	CUP	LN	beside 2	6.5	7			2			smooth thick patina			Limestone	
28	RCU	CUP	LN	beside 2	6.5	6			0.5			smooth thick patina			limestone	Pecked occurred in modern times, as indicated by no patina.
3	RCU	NCM	LN	Com. I	14	15	4		37	ground	smooth				limestone	Eroded at bottom.
30	RCU	CUP	LN		28	14			0.5			smooth thick patina			limestone	Questionable; eroded.
4	RCU	**NCM**	LN	Com. I	16		4			pecked	coarse ground				limestone	Oblong field stone wedged in mortar in recent times.
32	RCU	CUP	LN		7	5			2			rough			limestone	Identification debatable; eroded surface.
6	RCU	**NCM**	LN	Com. II, on boulder	17	16	3		20		coarse ground	coarse ground			limestone	Crack along the middle widened the NCM; was wider than usual.
6/1 .	RCU	CUP			6		4		2			Rough			limestone	Identification debatable; eroded CUP.
7	RCU	**NCM**	LN	W of S bedrock	12	12.5	5.5		27	fine ground	smooth	smooth			limestone	Hard stone at bottom reduced by flaked, pecked, ground of sides.
5	RCU	CUP		Com. II	4	5	5		0.5	pecked, ground	fine ground				limestone	
15	RCU	CUP			11				8		ground	pecked			limestone	Conical section due to hard stone at one side.
8	RCU	**NCM**	LN	Com. II, on oblong S bedrock	19		9		42	pecked , ground	fine ground	fine ground			limestone	Funnel rim; 2 cracks at bottom.
9	RCU	**WCM**	LN	Com. II	9.5		9				ground,	fine ground			limestone	Cracked in 2; radial striation.
10	RCU	CUP		Com. II	7	8	4		2.5			rough			limestone	Mostly eroded.
11`	RCU	**NCM**	LN	near monolith	16	16.5			31	pecked , ground	smooth	smooth			limestone	Horizontal cracks in 2 sides above bottom.
17	RCU	**NCM**	LN	Com. II	17.5	18.5	4		36	fine ground	fine ground				limestone	Hard stone at bottom reduced by flaked, pecked, ground of sides.
21	RCU	CUP	LN	beside 17	7	6.5			4.5	smooth	smooth, radial striation				limestone	Eroded, cracked on one side.
18	RCU	**NCM**	LN	Com. II	16	17	4		54		fine ground	smooth			limestone	Long narrow mortar; eroded rim; hole at bottom.
19	RCU	CUP	LN	Com. II	10.5		4		7		ground pecked				limestone	Slightly conical section; eroded.
20	RCU	**NCM**	LN	Com. II	19		5		.						limestone	Rim, upper part broken (like 33).

33	RCU	CUP	LN	on surface	19				13							limestone	
22	RCU	**NCM**	LN	narrow rock	13		4		.		fine ground					Limestone	Rim broken, eroded; 1 side cracked; lower part holed by l crack.
24	RCU	**NCM**	LN	Com. II	18		2		26		pecked, ground	fine ground				Limestone	Eroded rim; cracks on sides; end pierced; stone replace in hole.
Abu Salem 1	RCU	**NCM**	H	surrounded by 6 cups	14		4.5		21	fine ground	smooth	smooth					Side holed by karstic erosion.
1/2		CUP		beside 1	11	8	7		1.5			smooth				Limestone	
1/3		CUP		beside 1	13	10	11	5	2			smooth				limestone	
1/4		CUP		beside 1	13		9		1.5			smooth				limestone	
1/5		CUP		beside 1	11.5		11		1.5			smooth				limestone	
1/ 6		CUP		beside 1	14	13	9	10	2			smooth				limestone	
1/7		CUP		beside 1	12.5		4		2.5			smooth				limestone	
2	RCU	**NCM**	H	2/1	15		8		21	pecked, fine ground	fine ground	fine ground				limestone	
2/1	RCU	CUP		Str. 22	11.5		3		2			smooth, pecked				limestone	
Ramat Harif 1	RCU	**NCM**	H	50 m W of Str.	14		6		18	pecked	very smooth	very smooth				limestone	Some erosion on rim edge.
2	RCU	**NCM**	H	beside 1												limestone	.
3	RCU	**NCM**	H	part of wall Str.					30							limestone	Reduced hard stone at bottom, fine ground sides around stone.
Rosh Horesha 1	RCU	**NCM**	LN	10 m. E of MBI? Str.	18	17		6.5	26	pecked , ground	coarse ground					limestone	As 2.
1/1		CUP		10 m. E of MBI? Str.	9	8		5	3.5			fine ground				limestone	Oval cup; surface roughened by crumbled rock.
Saflulim 1	CB	**NCM**	**LN**	Com. I	20		19		42	rough	rough		47	41	31	limestone	57 narrow mortars are covered, could not identify as NCM or otherwise.
17	RCU	**NCM**	**LN**	Com. IV	15	13	3.5		42	coarse ground	fine ground	smooth					Straight sides, round bottom; lower side fine ground; smooth end.
18	RCU	**NCM**	**LN**	Com. IV	15	16	5		42	smooth	ground	ground				limestone	Section similar to mortar 16; one side eroded.
20	RCU	**NCM**	**LN**	Com. IV	17		4		33		pecked, coarse ground	ground				limestone	Bottom pierced into soft layer formation; pointed end.
21	RCU	**NCM**	**LN**	Com. IV	28		4		50	fine ground		fine ground				limestone	Wide mortar, bottom, round end; one side of rim missing.
26	RCU	**NCM**	**LN**	Com. IV	16.5	14.5	2		46	coarse ground	coarse ground	fine ground				limestone	Narrow mortar with long cylindrical shaft; one side mostly eroded.
48	RCU	**MU**	LN	Com. V	39	26			2			ground				limestone	2 cups at bottom.
55	RCU	**NCM**	LN	Com. VIII												limestone	
104	RCU	**MU**	LN	Com. V	16	19	4	12	3.5							limestone	
113	RCU	**MU**	LN	Com. V	21.5	15.5			5							limestone	
124	RCU	**NCM**	LN	Com. V	21		4		25							limestone	
172	RCU	**MU**	LN	Com. IX	27	12			3.5			rough	.	.	17	limestone	Eroded; fragment of wide irregular oval.
173	RCU	**NCM**	LN	Com. IX	20.5											limestone	
174	RCU	CUP	LN	Com. IX												limestone	
175	RCU	CUP	LN	Com. IX	10		17									limestone	
Romam 1-6	RCU	**6 NCMs**	H														Many other unexposed, possibly NCM.
Upper Besor VI 3	RCU	**MU**	N	Com. II	23	13			10		pecked, smooth					limestone	V-shaped groove from 3 to 5 used to drain rainwater (post-N).
1	RCU	**NCM**	LN	Com. I	23		4		30							limestone	Surrounded by circle of l stones.

6	RCU	**NCM**	LN*	Com. III	22						limestone	Partly exposed.
7	RCU	**NCM**	LN*	Com. III	23						limestone	Partly exposed.
I 8	RCU	**NCM**	LN*	Com. III							limestone	Partly exposed.
9	RCU	**NCM**	LN*	Com. III							limestone	Partly exposed.
10	RCU	**NCM**	LN*	Com. III							limestone	Partly exposed.
Wadi Mataha II 1	RCU	**NCM**	LN*	E edge of Exc.	15	1	72		ground	fine ground	sendstone	.
2	RCU	**NCM**	LN*	E edge of Exc.	25	2	30		ground	fine ground	sendstone	Long vertical grooves along the mortar sides due to use-wear of pestle on the sendstone sides.
3	RCU	**NCM**	LN*	E edge of Exc.	14		50		pecked	pecked	sendstone	.
4	RCU	**NCM**	LN*	E edge of Exc.	20			pecked		pecked	sendstone	.
5	RCU	**NCM**	LN*	E edge of Exc.							sendstone	.
