# Supplementary material for: Experimental Barley Flour Production in 12,500-Year-Old Rock-Cut Mortars in Southwestern Asia
Source: PLoS One. 2015 Jul 31;10(7):e0133306. doi: 10.1371/journal.pone.0133306 (PMC4521830; doi:10.1371/journal.pone.0133306)
Supplement: S1 Text — (DOCX) [file pone.0133306.s005.docx]

Supporting information **S1** **Text**

**Traditional unleavened bread and ancient barley groat meals**

Frikee, a traditional Arab common dish, is roasted unripe wheat grains and occasionally barley. Frikee ("rubbed" in Arabic) is prepared by roasting rather green ears on an open fire, drying them in the sun and peeling them by rubbing. The hardened grains, when ground into coarse groats and cooked, produce a gourmet dish with a delicious, smoky aroma and caramel flavor. During the Othman period and before, frikee was primarily made by the poor or during times of distress [1]. The dish called geres carmel in Biblical Hebrew (Lev 2, 14) seems to be identical to frikee and indicates that the dish was also a staple in ancient Israel. Ancient frikee was probably prepared from husked or partly peeled barley groats, called rifoth in Biblical Hebrew (Prov 27, 8), rosh (in Ugaritic) or arsanum in Akkadian (etymologically identified with the Talmudic 'arsan, Yerushalmi, Nedarim, 39, 3). The numerous flint blades found in Natufian sites is evidence of the harvesting of un-ripened barley with hafted sickles, in addition to the collecting of ripened grains for flour making. These findings may suggest that frikee was a common food beginning in the Early Natufian. Although unleavened bread was not found in Natufian sites, nor were hardly any plant remains found there due to poor preservation, a possible major product that could be prepared from flour in Late Epipaleolithic was unleavened bread. Fried or cooked cereal foodstuff food, like biscuits, pancakes and donuts could not be prepared without portable pottery vessel, nor pastries without oven. Reconstruction of our experimental bread was based on the preparation of Negev Bedouin. While flour can be eaten per se or mixed with water without de-husking the grains, the investment of the time and energy for de-husking wild barley for producing flour would not seem worthwhile for its direct consumption. It therefore seems plausible that the end product was bread (libe in Arabic; pers. obs. D.E.).

**Reference**

1. Azm AA. The importance and antiquity of frikkeh: a simple snack or a socio-economic indicator of decline and prosperity in the ancient Near East? In: Fairbairn AS, Wiess E, editors. From foragers to farmers. Oxford: Oxbow Books; 2009. pp. 112-116.
