# Supplementary material for: Experimental Barley Flour Production in 12,500-Year-Old Rock-Cut Mortars in Southwestern Asia
Source: PLoS One. 2015 Jul 31;10(7):e0133306. doi: 10.1371/journal.pone.0133306 (PMC4521830; doi:10.1371/journal.pone.0133306)
Supplement: S2 Text — (DOCX) [file pone.0133306.s006.docx]

Supporting information **S2 Text**

**Technical analysis**

To peel barley grains, shearing forces must be created, such as holding the grain and rubbing the peel parallel to its surface. In contemporary grain processing machinery, two parallel planes holding the grains move fast in opposite directions. Contact of each grain with the fast moving surface creates a frictional force on the peel along its plane. The inertia of the grain center of mass reacts in the opposite direction and the pair of forces "shears" the peel.

The Natufians narrow conical mortars frequently contained three parts: a funnel shaped upper part, a narrow conical mortar and a cylindrical narrow shaft at the end of the mortar, sometimes pierced at the bottom (S1C and S1D Figs.). The morphological and technological analysis of the mortars shows that only the middle part – the narrow conical mortar – was involved in its prehistoric use [[1](#_ENREF_1), [2](#_ENREF_2)]. The funnel upper part, lacking wear of use, was found only in the deeper narrow conical mortars (more the 35-40 cm depth) and was probably the result of widening the mortar by carving, enabling the fabrication of the deep narrow mortar. The shaft shape and markings seem to have resulted from intensive and long duration vertical and radial motions by a pointed pestle, sometime piercing the bottom. The radius of the shaft enabled us to reconstruct the radius of the pestle’s pointed end.

A pointed conical wooden pestle pressed into the conical hole, forces the loaded grains to move upwards. The mass of grains behaves like a “fluid.” If the pestle moves fast enough, the flow of grains is “turbulent.” Frictional forces are created between neighboring grains, between the grains and the inner stone walls, as well as between the grains and the pestle. Since the sharp point of the pestle limits crushing at the bottom, repeated blows of the pestle eventually peel the grains. The process can be improved by optimizing the pestle material, affecting friction; the pestle shape and diameter, affecting the gap and the upward flow of grains; and the speed and type of pestle motion (parallel/elliptical/horizontally revolving, etc.). Mechanical properties of the grain – such as shape, strength of peel-grain adherence, grain hardness, friction between the grains and between the grains and the wooden pestle and stone – strongly affect efficiency. We can assume that, save for the stone material and grain type, the Natufians optimized their tools. The fact that, without optimization, we have successfully achieved clean grains ready for bread making is amazing! Thus, this tool was a peeling machine way before peeling machines were born.

**Reference**

1. Eitam D. Late Epipaleolithic rock-cut installations and ground stone tools in the Southern Levant: methodology and typology. Paléorient. 2009;31: 77-104. 2. Eitam D. Archaeo-industry of the Natufian culture: Late Epipalaeolithic rock-cut installations and groundstone tools in the Southern Levant. Unpublished PhD dissertation, Jerusalem: Hebrew Univ; 2013 (Hebrew).
